# Bibliometric Analysis of Chronic Traumatic Encephalopathy Research from 1999 to 2019

**DOI:** 10.3390/ijerph17155411

**Published:** 2020-07-28

**Authors:** Bote Qi, Shuting Jin, Hongsheng Qian, Yu Zou

**Affiliations:** 1 Department of Sport and Exercise Science, College of Education, Zhejiang University, 886 Yuhangtang Road, Hangzhou 310058, China; qibote@zju.edu.cn; 2 College of Computer Science and Technology, Zhejiang University, 886 Yuhangtang Road, Hangzhou 310058, China; stefanie.jinst@gmail.com; 3 College of Physical Education, Central China Normal University, 152 Luoyu Avenue, Wuhan 430079, China; qianhsheng@126.com

**Keywords:** bibliometrics, CiteSpace, VOSviewer, chronic traumatic encephalopathy

## Abstract

Research on chronic traumatic encephalopathy (CTE) has increased over the past two decades. However, few studies have statistically analyzed these publications. In this work, we conducted a bibliometric analysis of studies on CTE to track research trends and highlight current research hotspots. Relevant original articles were obtained from the Web of Science Core Collection database between 1999 and 2019. CiteSpace and VOSviewer software were used to perform analysis and visualization of scientific productivity and emerging trends. Our results show that the publications related to CTE dramatically increased from four publications in 1999 to 160 publications in 2019. The United States dominated this field with 732 publications (75.934%), followed by Canada with 88 publications (9.129%). Most of related publications were published in the journals with a focus on molecular biology, immunology, neurology, sports and ophthalmology, as represented by the dual-map overlay. A total of 11 major clusters were explored based on the reference co-citation analysis. In addition, three predominant research topics were summarized by clustering high-frequency keywords: epidemiological, clinical and pathological studies. The research frontiers were the diagnosis of diseases using new neuroimaging techniques, and the investigation of the molecular mechanism of tau aggregation. This study provides researchers with valuable guidance in the selection of research topics.

## 1. Introduction

Chronic traumatic encephalopathy (CTE) is a progressive neurodegeneration that results from repeated head trauma, and it is characterized by the widespread deposition of hyperphosphorylated tau (p-tau) as neurofibrillary tangles [1,2,3,4]. CTE was originally reported in 1928 by Martland, who described the clinical aspects of a progressive neurological deterioration (punch drunk) that occurred after repetitive brain trauma in boxers [5]. Milspaugh described the same syndrome in 1937 and introduced the term “Dementia Pugilistica” [6]. With the development of research, “Chronic Traumatic Encephalopathy” was widely used to describe the disease. CTE occurs in a wide range of contact sports (boxing, rugby, wrestling, football and Mixed Martial Arts) [7,8,9,10,11]. A retrospective cohort study revealed varying incidence of CTE in players of different sports, with an overall incidence of 9% [12]. Furthermore, additional large groups of individuals prone to repetitive head trauma such as military personnel [13,14] and domestic abuse victims [15], can also be at risk of CTE. The clinical symptoms of CTE include memory impairment and executive dysfunction, sleep disturbances, behavioral and personality changes, and the onset of these symptoms is usually 8–10 years after experiencing repetitive mild traumatic brain injury [8]. More severe neurological changes emerge as the disease progresses including dementia, gait and speech abnormalities, and parkinsonism [16]. Three-time world heavyweight boxing champion, Muhammad Ali, experienced repeated blows to the head throughout his career. Later in life he developed symptoms related to Parkinson’s disease. However, no disease-modifying therapies of CTE currently exist, and diagnosis requires an autopsy [17]. Therefore, in order to protect the health rights of athletes, relevant international sports organizations and scientific research institutions have proposed a series of preventive measures and recommended that injured athletes should follow strict “return to play” guidelines to effectively prevent CTE [8].

An increasing number of researchers have shifted their focus to the field of CTE, especially for the epidemiological, clinical and pathological studies over the last two decades. The number of articles related to CTE has been growing rapidly. Therefore, evaluating the quantitative and qualitative value of these articles from a scientific perspective is conducive for advancing research in the field of CTE. By utilizing the scientific mapping tools (CiteSpace and VOSviewer), this study provides researchers working on CTE with an in-depth and broad view of the underlying knowledge structure, and progressive evolution of this emerging interdisciplinary field.

## 2. Materials and Methods

### 2.1. Data Collection

**Figure 1:**
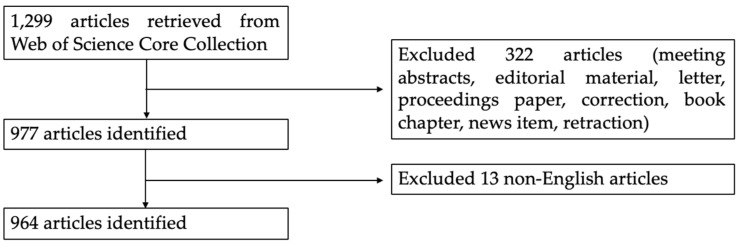
A flowchart representing retrieval strategies for chronic traumatic encephalopathy (CTE) articles from the Web of Science Core Collection (WoSCC) database and the inclusion criteria for the study.

All articles were retrieved from Web of Science Core Collection (WoSCC) on 8 February 2020. We performed all searches in one day to avoid database update bias. WoSCC contains relatively reliable data and provides extensive information for analysis [18]. The following methods were used to search for articles published from 1999 to 2019: Enter the Topic words = (“chronic traumatic encephalopathy” or “punch drunk syndrome” or “chronic traumatic brain injury” or “dementia pugilistica” or “chronic head trauma”). In WoSCC, topic search is the most useful when searching for a subject. This option enables researchers to search all the subject-related parts of the records (title, abstract, keywords) in the database. The present study only selected articles or reviews for analyses based on the relevance of the paper, and the language of publications was limited to English. The data extraction was performed by using the Web of Science Refine Results panel in WoSCC. We first selected the check box of “Document type”/ “Languages” on the list and chose “ARTICLE”/“ENGLISH”, and then clicked the Refine button to view those results. All downloaded data was independently checked by two researchers (Bote Qi and Hongsheng Qian). The detailed data retrieval strategies and inclusion criteria for this study are summarized in Figure 1.

### 2.2. Data Analysis

Article information was extracted from the WoSCC database including authors, titles, abstracts, organizations, countries, journals, keywords, and references, which was subsequently saved in the “Plain Text” format. We used three software tools (CiteSpace 5.6.R2, VOSviewer 1.6.13 and Microsoft Excel 2016) to analyze search results and to assess the different aspects of information obtained from the WoSCC database. This software tools have been widely used in previous bibliometric studies [19,20,21]. CiteSpace 5.6.R2 software (Drexel University, Philadelphia, PA, USA) [22,23] was used for visualization of collaborative networks, reference citations and analyses of research hotspots. VOSviewer 1.6.13 software (Leiden University, Leiden, The Netherlands) was used to analyze clusters of keywords [24]. Microsoft Excel 2016 was used to predict the future trends of CTE publications. The equation of the prediction model was as follows: f(x) = ax^3^ + bx^2^ + cx + d, in which x represented the publication year, and f(x) represented the cumulative number of publications. In this way, we effectively captured the current status, emerging trends, and recent developments in the research of CTE.

## 3. Results

### 3.1. Publication Outputs and Development Trend

**Figure 2:**
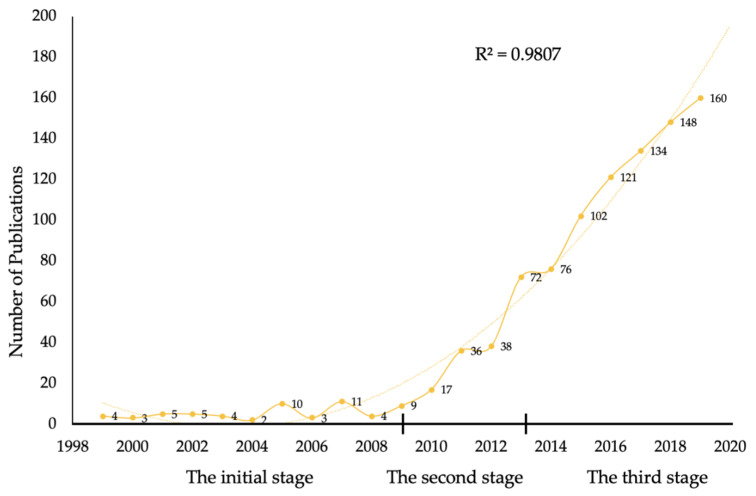
The output of publications and growth prediction of CTE research. The number of publications from 1999 to 2019 are represented by the solid line; the dashed line represents the predicted curve, R^2^ = 0.9807.

A total of 964 articles matched the retrieval criteria and were used for further analysis. The distribution of annual publications and the growth trend of the model-fitting curve are presented in Figure 2. Quantitative analysis revealed that global research on CTE has rapidly increased in the past 20 years, with articles increasing rapidly from four in 1999 to 160 in 2019. Research on CTE has been progressive, indicating that CTE has become a subject of interest in field of neurosciences. Moreover, a growth trend model (R^2^ = 0.9807) predicted that 195 articles on CTE will be published by 2020, signifying the growing interest of researchers in this field.

Research on CTE was divided into three stages: the initial stage, second stage and third stage. The years 2009 and 2013 were the key turning points. In 2009, McKee et al. [8] reviewed the literature for 47 CTE cases and established that patients with CTE had extensive tau-immunoreactive neurofibrillary tangles, astrocyte tangles, and spindle-shaped and threadlike neurites in the brain. In 2013, McKee et al. [16] indicated that CTE had four crucial pathological stages. The severity spectrum of hyperphosphorylated tau pathology ranged from the focal perivascular epicentres of neurofibrillary tangles in the frontal neocortex to severe tauopathy affecting extensive regions of the brain. The two studies by McKee and colleagues significantly contributed to the advancement of CTE research.

### 3.2. Analysis of Countries and Institutions

**Figure 3:**
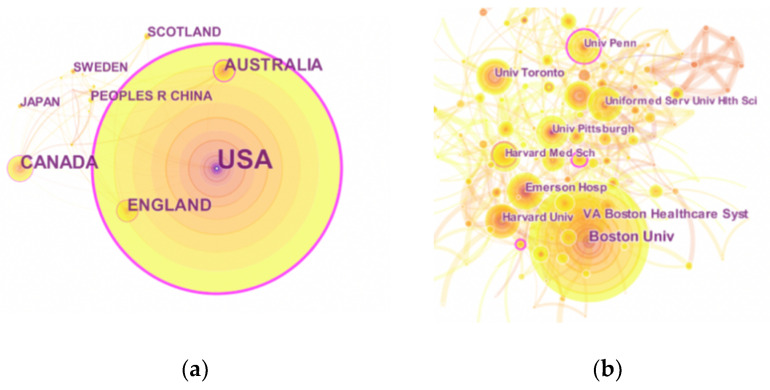
The distribution of countries and institutions. The nodes represent the countries and institutions; the color depth and size of the circle are positively correlated to the number of posts. The thickness of the curved connecting lines represents the strength of collaboration in the countries and institutions. (**a**) Map of countries with publications on CTE. (**b**) Map of institutions with publications on CTE.

The United States had the highest number of publications, 732, and the highest centrality (0.82) (Table 1). A total of 1235 institutions contributed to CTE research, of which nine of the top 10 institutions were from the United States. This shows that the United States has conducted extensive research on CTE, and the credibility of the publications in this field of research has been recognized. Among these active institutions, Boston University has the highest number of publications. Boston University’s CTE Center is an independent academic research lab located at Boston University School of Medicine. It was established in 1996 and conducted many high-impact, innovative studies on CTE [7,8]. In addition, a network map was generated using CiteSpace V to visually present the connections in the countries and institutions that have contributed to research in the CTE field. The United States was leading in CTE research (Figure 3). The abundance of connection lines indicates that there was extensive cooperation between institutions, and that there was a closer connection between institutions that published fewer articles.

### 3.3. Journal Analysis

**Figure 4:**
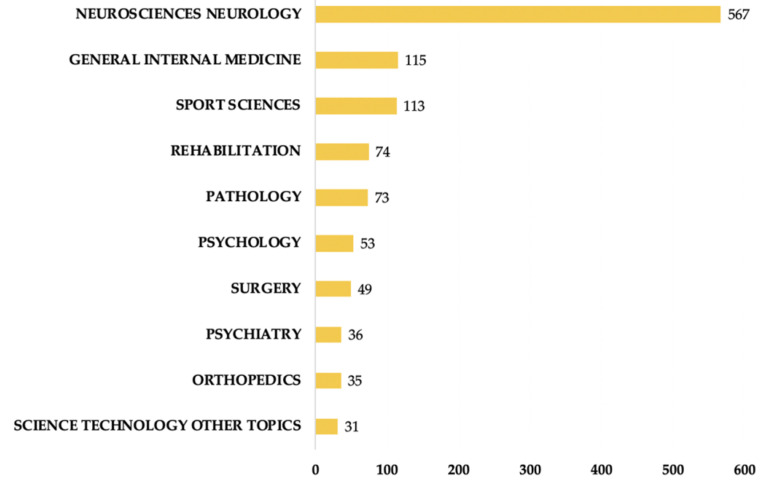
Ranking of top 10 active research areas on CTE from 1999 to 2019.

**Figure 5:**
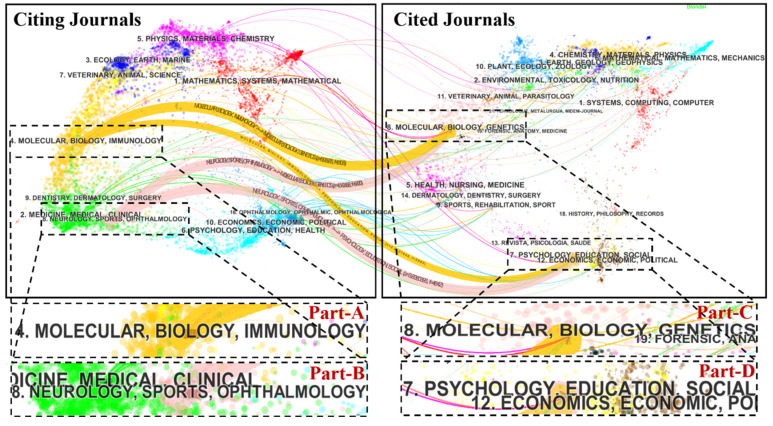
A dual-map overlay of journals that published work related to CTE. A presentation of citation paths at a disciplinary level on a dual-map overlay. The width of the paths is proportional to the z-score-scale citation frequency.

A total of 375 scholarly journals published articles on CTE research. The top 10 journals are presented in Table 2. The Journal of Neurotrauma (IF 2018 = 3.754) published the highest number of articles (64 publications, 6.639%), followed by Frontiers in Neurology (IF 2018 = 2.635), the Journal of Alzheimer’s Disease (IF 2018 = 3.517) and the Journal of Neuropathology and Experimental Neurology (IF 2018 = 3.46). The top 10 research hotspots largely published by the leading journals are presented in (Figure 4). Neurosciences and neurology ranked first among the key research hotspots for CTE research, with 567 articles. Other research hotspots included general internal medicine, sport sciences and rehabilitation research. Figure 5 shows a dual-map overlay of the number of articles with reference to the type or focus of the journal. The labels on the map represent the research subjects covered by the journals. The citing journals are on the left side of the map whereas the cited journals are on the right side of the map. Overall, published articles targeted journals in the fields of molecular biology, immunology (Part-A journals), and neurology, sports and ophthalmology (Part-B journals), whereas the most cited papers were published in the journals of molecular biology and genetics (Part-C), and psychology, education and social (Part-D). As the cited journals provide the knowledge base of the citing journals, these shifting trajectories showed that the disciplinary center of the journals moved from genetics, psychology, education and social to immunology, neurology, sports and ophthalmology.

### 3.4. Analysis of Authors and Co-cited Authors

**Figure 6:**
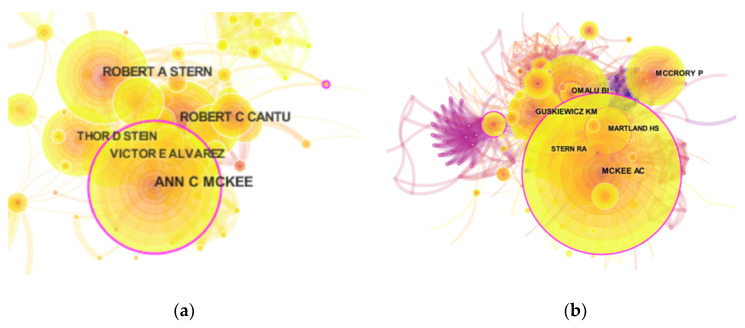
(**a**) Map of authors active in CTE research. (**b**) Map of co-cited authors active in CTE research. The nodes represent the authors, and the color depth and shape of the circles are positively correlated to the number of posts. The thickness of the connecting lines represents the strength of collaboration.

More than 3792 authors contributed to CTE research. The top 10 authors involved in CTE research are listed in Table 3. These authors have collectively published a total of 295 papers, accounting for 30.602% of all published papers on CTE research. Figure 6a shows the degree of cooperation between authors. McKee AC (63 publications) was identified as the most active author in the field of CTE research with 63 publications, followed by Stern RA with 45 publications and Cantu RC with 41 publications. Co-cited author was also a key criterion for assessing the contribution of researchers. The top three co-cited authors were McKee AC, Omalu BI, and McCrory P (Figure 6b). The top 10 most co-cited references are listed in Table 3. These publications laid a foundation and promoted the development of research in the CTE field. The top three co-cited references were authored by McKee AC, and the articles published in the Brain Journal had the highest number of citations (394 citations). This implies that the achievements of McKee AC in this field are highly authoritative.

### 3.5. Analysis of Reference Co-Citation

**Figure 7:**
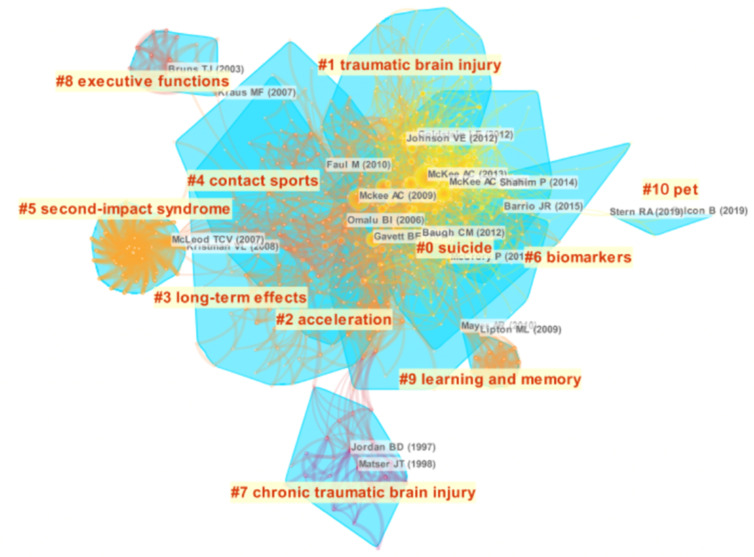
The cluster view of the knowledge map based on reference co-citation analysis (RCA) of the CTE field from 1999 to 2019.

**Figure 8:**
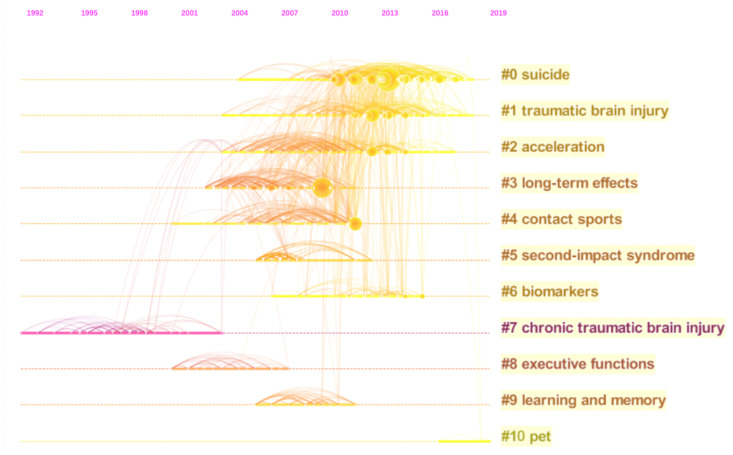
The timeline view of the knowledge map based on RCA of the CTE field from 1999 to 2019. This view represents the appearance of eleven clusters at different time points and time spans.

Reference co-citation analysis (RCA) is one of the core indices of bibliometrics, which explores the co-citation relevance between articles and generalizes data to create major clusters. RCA is usually used to explore research hotspots in a given academic field [25,26]. RCA was performed to generalize clusters and construct mapping knowledge domains of clusters (Figure 7) and timeline (Figure 8) views. With reference to the RCA (Figure 7), the articles on CTE research published between 1999 and 2019 were clustered into 11 major research hotspots. Each cluster highlighted the citation index, research field and key literature groups within a period of time, showing a distinct specialty or thematic concentration. The value of modularity (Q) was 0.6607, and the mean silhouette value was 0.2973. The largest cluster (#0) out of the 11 clusters was associated with suicide, followed by cluster #1 (traumatic brain injury) and cluster #2 (acceleration). The timeline view for all clusters, which indicated the time span and research progress in the development and evolution of each cluster sub-domain, is presented in Figure 8.

### 3.6. Analysis of Keywords

**Figure 9:**
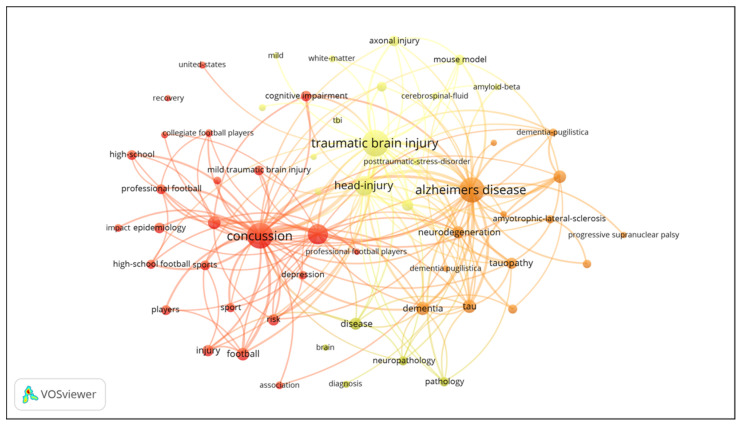
The cluster view of high frequency keywords in the CTE research field from 1999 to 2019. Each color in the figure represents a category, and keywords with the same color belong to the same cluster. The keyword “chronic traumatic encephalopathy” was excluded from our search for an improved understanding.

**Figure 10:**
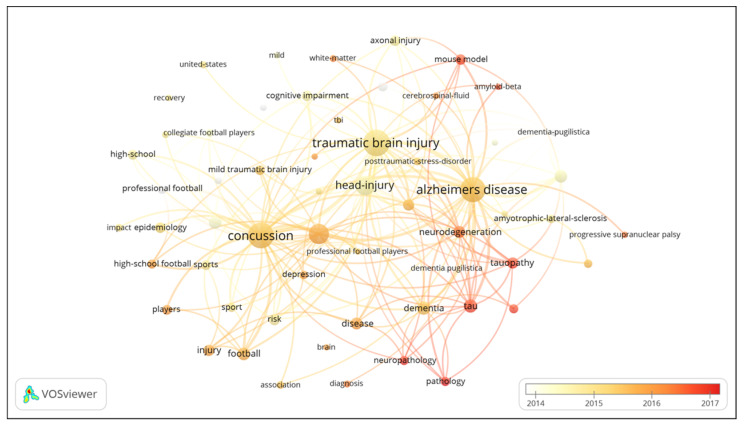
Distribution of keywords based on the time of appearance. Keywords marked by the circles in different colors (yellow early; red later).

A cluster visualization of high frequency keywords was performed using VOSviewer to understand the research topics in a more comprehensive manner [24]. The results revealed that there were 3661 keywords in 964 articles, and 105 keywords appeared 18 times or more. The co-occurrence network map of keywords reflects the static structure of CTE research, and shows the clustering of three major themes (epidemiological, clinical and pathological studies) in the field of CTE research (Figure 9). The frequencies of primary keywords for the cluster of epidemiological studies were as follows: concussion (281 times), brain-injury (181 times), football (88 times) and players (59 times). The research direction of epidemiological studies mainly focuses on cross-sectional and longitudinal research. The frequencies of primary keywords for the clinical studies cluster were as follows: diffuse axonal injury (59 times), rehabilitation (33 times), performance (26 times) and memory (20 times). The frequencies of primary keywords for the cluster of pathological studies were as follows: tau (90 times), neurofibrillary tangles (85 times), tauopathy (71 times), and amyotrophic-lateral-sclerosis (45 times). The clinical and pathological studies separately focus on case-study and experimental research. The VOSviewer applied colors to keywords based on when they appeared in a journal (Figure 10). A keyword can serve as an important index in reflecting research hotspots at a given time, and help to predict new frontier topics. Research keywords such as “tau”, “tauopathy” and “neuroinflammation” have appeared in the cluster for pathological studies over the last few years.

## 4. Discussion

### 4.1. Research Hotspots

The research hotspots of the epidemiological, clinical and pathological studies in the fields of CTE over the last two decades were summarized based on the results of reference co-citation and keyword analyses. Analyzing the incidence of CTE in different sports and preventing the prevalence of CTE are the principal research directions in the field of epidemiology. A retrospective cohort study revealed that athletes participating in different sports events had varying incidences of CTE, with an overall incidence of 9% [12]. CTE is widespread among athletes involved in contact sports such as rugby and football. One of the most comprehensive studies conducted on CTE indicated that CTE was neuropathologically diagnosed in 177 (87%) of the 202 deceased former rugby players [7]. A study involving a 30-year follow-up of retired football players (42.8%) revealed that six out of 14 cases presented clinical characteristics corresponding to CTE [27]. Moreover, the duration of vigorous contact exercise demonstrated a strong dose–response relationship with neuropathology of CTE. The longer the duration of exercise, the higher the risk of illness [28]. The high prevalence of CTE among professional athletes has attracted the attention of researchers in the fields of sports science and sports medicine around the world. The relevant international sports organizations and scientific research institutions have proposed a series of preventive measures to reduce the incidence of CTE. One of the most important measures is to reduce the repeated blows to the head. According to statistics, every year an estimated 42 million people worldwide suffer a mild traumatic brain injury (MTBI) or concussion [29]. Modern tracking devices have found that a football player suffered thousands of sub-concussive hits to the head during a single season [30]. Repetitive MTBI is closely related to the increased risk of CTE [15]. Thus, the International Rugby Commission enacted laws to prevent head contact incidents in rugby [31]; the Concussion in Sport Group has provided medical education and raised awareness on concussion through the four Concussion Consensus Conferences and Statements [32]. In addition, CTE also occurred in military veterans. Statistics showed that more than 300,000 service members and veterans have sustained at least one blast- and/or impact-related traumatic brain injury because of the widespread use of conventional and improvised explosive devices (IED) in the conflicts in Iraq and Afghanistan [33]. Goldstein’s study suggested that blast exposure may increase risk for later development of CTE and associated neurobehavioral sequelae [14]. Mckee et al. reported that of the 110 cases neuropathologically diagnosed with CTE at the Boston VA TBI Brain Bank, CTE has been diagnosed in 23 veterans [34].

Evaluating clinical symptoms at different stages and establishing the corresponding clinical diagnostic procedures are the two crucial research directions in the field of clinical research. According to previous studies [35,36], the clinical symptoms of CTE were divided into four stages: (1) stage I was characterized by headache and attention deficit, (2) individuals with stage II CTE experienced depression and mood swings, explosivity, loss of attention and concentration, headache and short-term memory loss, (3) most individuals with stage III CTE exhibited cognitive impairment, executive dysfunction, loss of attention and concentration, depression, explosivity and visuospatial abnormalities, and indicated irreversible brain damage, (4) individuals with stage IV CTE had dementia with profound short-term memory loss, executive dysfunction, attention and concentration loss, and aggression. Most of the individuals with stage IV CTE also exhibited paranoia, depression, impulsivity and visuospatial abnormalities, and even suicidal tendencies. A combination of the clinical symptoms at the different stages of CTE and formulation of corresponding clinical diagnostic procedures and standards is essential in providing guidance during screening at the early stages of the disease. Montenigro et al. proposed a fundamental process for the clinical diagnosis of CTE by summarizing the clinical characteristics of 202 cases of CTE, which included five general criteria and three core clinical features [37]. The proposal provided a preliminary conception and directional guidance for CTE diagnostic criteria. In addition, Victoroff and Jordan et al. proposed corresponding clinical diagnostic methods, but their effectiveness in clinical application is yet to be evaluated [38,39].

Studying the pathological characteristics of CTE at different stages and establishing the corresponding diagnostic criteria for neuropathology are the two central research hotspots in the field of pathology. The different pathological stages of CTE have distinct pathological characteristics [16,40,41]. In patients with mild CTE, the focal perivascular epicenters of neurofibrillary tangles (NFTs) and astrocytic tangles (ATs) were found clustered at the depths of the cortical sulci. In patients with severe CTE, extreme tauopathy affects large areas of the brain. Other abnormalities exhibited during severe CTE include abnormal deposits of phosphorylated TAR DNA-binding protein of 43 kDa (TDP-43), a protein that occasionally colocalizes with p-tau, varying degrees of Aβ pathology, axonal dystrophy and neuroinflammation [42]. Establishing corresponding diagnostic criteria for neuropathology has become the focus of pathological studies in the last few years. McKee et al. proposed supportive neuropathological diagnostic criteria for CTE, which included five supporting pathological features associated with phosphorylated tau (Table 4) [43]. The current research efforts, which are guided by pathological findings, are focused on developing biomarkers for diagnosing CTE and effective methods for treating the disease.

### 4.2. Research Frontiers

The timeline view of the knowledge map indicates that cluster “#10 PET” is the current research frontier in the CTE research field (Figure 8). The cluster “#10 PET” denotes positron emission tomography (PET). PET is a new imaging technology which can reveal the metabolism of biomolecules, the activity of receptors and neurotransmitters in vivo. This technology creates a three-dimensional image by detecting the concentration of tracer injected into the body. PET facilitates intuitive understanding of various physiological or pathological metabolic changes in the human body. Presently, the most commonly used PET imaging agent is [F-18] FDDNP [44,45,46,47]. [F-18] FDDNP-PET can visualize and quantify the regional presence of tau deposits in areas of neural aggregates in the living human brain of a suspected CTE case, and generates useful information on the underlying mechanisms of disease staging [48]. Furthermore, a study has demonstrated that [F-18] FDDNP-PET can identify CTE substrates in living patients, not only based on the presence or absence of protein lesions, but also on the identification of differential and selective topographic vulnerability unique to CTE [49]. PET may serve as a method for premorbid identification of neurodegeneration in athletes participating in contact sports in the future.

The distribution of keywords with time demonstrates that “tau protein”, “tauopathy” and “pathology” are the keywords of research frontiers in the field of CTE (Figure 10). This indicates that analyzing the relationship between “tau protein”, “tauopathy” and CTE from the perspective of “pathology” substantially contributes to the advancement of CTE research. Tau protein is a highly soluble microtubule-associated protein that is primarily distributed in neurons of the central nervous system [50,51]. Aggregation of tau into insoluble filaments is the defining pathological hallmark of tauopathies (including chronic traumatic encephalopathy, Alzheimer’s disease and corticobasal degeneration). Tau as an intrinsically disordered protein, is highly flexible and has variable conformations, making it difficult to perform structural analysis. The fibril structure of tau proteins and the atomic models of tubulin-bound tau have been elucidated with the development of experimental methods such as Cryo-SEM, SSNMR and X-ray in the recent years [52,53,54]. In 2019, Falcon et al. first described the specific characteristics of the molecular structure of tau protein in the brain of patients with CTE and revealed their detailed fibril structure models, which greatly enhanced understanding of the molecular mechanism of CTE [17]. Future studies (in vivo and in vitro) will focus on investigating the underlying mechanism of tau aggregation to further elucidate the pathogenesis of CTE.

### 4.3. Limitations

The present study had a few limitations. First, the data sources analyzed in our research were limited to the WoSCC database, and we did not include data from other relevant search engines (e.g., Embase, Medline and Scopus). Thus, the identified articles may not fully represent all CTE research. However, as one of the most comprehensive databases in the world, the WoSCC database has recognized the quality of its papers, and the data retrieved from this database is very suitable for CiteSpace and VOSviewer to carry out bibliometric analysis. Second, we only selected articles published in English, thereby resulting in language bias. Nonetheless, English remains the most widely used language for publishing academic articles.

## 5. Conclusions

A total of 964 articles on CTE research published between 1999 and 2019 were retrieved from the WoSCC database. The number of publications, key institutions and countries, published journals, primary authors, and cooperative networks were systematically analyzed using hybrid analysis and visualization technologies (CiteSpace and VOSviewer). The analysis of co-occurrence networks provides researchers with information about potential collaboration opportunities with other institutions and researchers. Bibliometric analyses also reveal the current research hotspots and research frontiers in an objective and comprehensive manner, thus indicating the retrospective view of CTE and providing valuable guidance for researchers in the selection of research topics.

## Figures and Tables

**Table 1:** Ranking of top 10 active countries and institutions in the field of CTE research from 1999 to 2019.

Rank	Country	Counts	Centrality	Institution	Counts	Centrality
1	United States	732	0.82	Boston University	114	0.12
2	Canada	88	0.09	VA Boston Healthcare System	48	0.04
3	England	72	0.17	Emerson Hospital	40	0.10
4	Australia	69	0.06	University of Toronto	38	0.05
5	Scotland	28	0.01	Harvard University	37	0.06
6	Japan	23	0.02	University of Pennsylvania	35	0.11
7	China	21	0.06	Uniformed Services University HLTH SCI	33	0.02
8	Germany	20	0.01	University of Pittsburgh	33	0.09
9	Ireland	20	0.09	Harvard Medical School	29	0.04
10	Sweden	17	0.00	Vanderbilt University	27	0.03
	Others	73		Others	1094	

**Table 2:** Ranking of top 10 active journals that published articles on CTE research from 1999 to 2019.

Ranking	Journal	Country	Count	Percentage (%)	IF 2019
1	Journal of Neurotrauma	United States	64	6.639	3.793
2	Frontiers in Neurology	Switzerland	24	2.490	2.889
3	Journal of Alzheimer’s Disease	The Netherlands	22	2.282	3.909
4	Journal of Neuropathology and Experimental Neurology	United States	21	2.178	2.923
5	Acta Neuropathologica	Germany	20	2.075	14.251
6	Brain Injury	England	19	1.971	1.69
7	British journal of Sports Medicine	England	18	1.867	12.022
8	Journal of Head Trauma Rehabilitation	United States	15	1.556	2.814
9	American Journal of Sports Medicine	United States	13	1.349	5.81
10	Acta Neuropathologica Communications	United Kingdom	12	1.245	6.27
	Others		736	76.349	

**Table 3:** Ranking of top 10 authors, co-cited authors, and co-cited references in the field of CTE from 1999 to 2019.

Rank	Author	Counts	Co-cited Author	Counts	Co-cited Reference	Count
1	McKee AC	59	McKee AC	648	McKee AC, 2013, Brain, V136, P43	394
2	Stern RA	42	Omalu BI	328	McKee AC, 2009, J Neuropath Exp Neur, V68, P709	334
3	Cantu RC	37	McCrory P	269	McKee AC, 2016, Acta Neuropathol, V131, P75	169
4	Stein TD	32	Guskiewicz KM	253	Gavett BE, 2011, Clin Sport Med, V30, P179	161
5	Alvarez VE	26	Stern RA	243	Goldstein LE, 2012, Sci Transl Med, V4, P0	150
6	Alosco ML	23	Martland HS	211	Stern RA, 2013, Neurology, V81, P1122	147
7	Tripodis Y	22	Omalu B	201	McKee AC, 2010, J Neuropath Exp Neur, V69, P918	145
8	Nowinski CJ	19	Jordan BD	199	Omalu B, 2011, Neurosurgery, V69, P173	142
9	Daneshvar DH	19	Corsellis JA	198	Stern RA, 2011, PM & R, V3, P0	120
10	Bauhg CM	16	Gavett BE	195	Baugh CM, 2012, Brain Imaging Behav, V6, P244	107
	Others	1115	Others	5725	Others	4357

**Table 4:** Preliminary National Institute of Neurological Disorders and Stroke (NINDS) criteria for the pathological diagnosis of CTE [43].

	Supportive Neuropathological Features of CTE
1	Abnormal p-tau immunoreactive pretangles and NFTs preferentially affecting superficial layers (layers II–III), in contrast to layers III and V as in AD
2	In the hippocampus, pretangles, NFTs or extracellular tangles preferentially affecting CA2 and pretangles and prominent proximal dendritic swellings in CA4. These regional p-tau pathologies differ from the preferential involvement of CA1 and subiculum found in AD
3	Abnormal p-tau immunoreactive neuronal and astrocytic aggregates in subcortical nuclei, including the mammillary bodies and other hypothalamic nuclei, amygdala, nucleus accumbens, thalamus, midbrain tegmentum, and isodendritic core
4	p-Tau immunoreactive thorny astrocytes at the glial limitans most commonly found in the subpial and periventricular regions
5	p-Tau immunoreactive large grain-like and dot-like structures
